# Immobilization of β-Galactosidases on the *Lactobacillus* Cell Surface Using the Peptidoglycan-Binding Motif LysM

**DOI:** 10.3390/catal9050443

**Published:** 2019-05

**Authors:** Mai-Lan Pham, Anh-Minh Tran, Suwapat Kittibunchakul, Tien-Thanh Nguyen, Geir Mathiesen, Thu-Ha Nguyen

**Affiliations:** 1 Food Biotechnology Laboratory, Department of Food Science and Technology, BOKU-University of Natural Resources and Life Sciences, A-1190 Vienna, Austria; 2 Department of Biology, Faculty of Fundamental Sciences, Ho Chi Minh City University of Medicine and Pharmacy, 217 Hong Bang, Ho Chi Minh City, Vietnam; 3 School of Biotechnology and Food Technology, Hanoi University of Science and Technology, 1 Dai Co Viet, Hanoi, Vietnam; 4 Faculty of Chemistry, Biotechnology and Food Science, Norwegian University of Life Sciences (NMBU), N-1432 Ås, Norway

**Keywords:** *Lactobacillus*, β-galactosidase, immobilization, cell surface display, LysM domains

## Abstract

Lysin motif (LysM) domains are found in many bacterial peptidoglycan hydrolases. They can bind non-covalently to peptidoglycan and have been employed to display heterologous proteins on the bacterial cell surface. In this study, we aimed to use a single LysM domain derived from a putative extracellular transglycosylase Lp_3014 of *Lactobacillus plantarum* WCFS1 to display two different lactobacillal β-galactosidases, the heterodimeric LacLM-type from *Lactobacillus reuteri* and the homodimeric LacZ-type from *Lactobacillus delbrueckii* subsp. *bulgaricus,* on the cell surface of different *Lactobacillus* spp. The β-galactosidases were fused with the LysM domain and the fusion proteins, LysM-LacLMLreu and LysM-LacZLbul, were successfully expressed in *Escherichia coli* and subsequently displayed on the cell surface of *L. plantarum* WCFS1. β-Galactosidase activities obtained for *L. plantarum* displaying cells were 179 and 1153 U per g dry cell weight, or the amounts of active surface-anchored β-galactosidase were 0.99 and 4.61 mg per g dry cell weight for LysM-LacLMLreu and LysM-LacZLbul, respectively. LysM-LacZLbul was also displayed on the cell surface of other *Lactobacillus* spp. including *L. delbrueckii* subsp. *bulgaricus, L. casei* and *L. helveticus,* however *L. plantarum* is shown to be the best among *Lactobacillus* spp. tested for surface display of fusion LysM-LacZLbul, both with respect to the immobilization yield as well as the amount of active surface-anchored enzyme. The immobilized fusion LysM-β-galactosidases are catalytically efficient and can be reused for several repeated rounds of lactose conversion. This approach, with the β-galactosidases being displayed on the cell surface of non-genetically modified food-grade organisms, shows potential for applications of these immobilized enzymes in the synthesis of prebiotic galacto-oligosaccharides.

## 1 Introduction

β-Galactosidases catalyze the hydrolysis and transgalactosylation of β-D-galactopyranosides (such as lactose) [1–3] and are found widespread in nature. They catalyze the cleavage of lactose (or related compounds) in their hydrolysis mode and are thus used in the dairy industry to remove lactose from various products. An attractive biocatalytic application is found in the transgalactosylation potential of these enzymes, which is based on their catalytic mechanism [1,4]. β-Galactosidases can be obtained from different sources including microorganisms, plants and animals, however microbial sources of β-galactosidase are of great biotechnological interest because of easier handling, higher multiplication rates, and production yield. Recently, a number of studies have focused on the use of the genus *Lactobacillus* for the production and characterization of β-galactosidases, including the enzymes from *L. reuteri, L. acidophilus, L. helveticus, L. plantarum, L. sakei, L. pentosus, L. bulgaricus, L. fermentum, L. crispatus* [5–15]. β-Galactosidases from *Lactobacillus* species are different at molecular organization [6,8,10,12,16]. The predominant glycoside hydrolase family 2 (GH2) β-galactosidases found in lactobacilli are of the LacLM type, which are heterodimeric proteins encoded by the two overlapping genes, *lacL* and *lacM,* including *lacLM* from *L. reuteri* [16], *L. acidophilus* [6], *L. helveticus* [7], *L. pentosus* [11], *L. plantarum* [8], and *L. sakei* [10]. Di- or oligomeric GH2 β-galactosidases of the LacZ type, encoded by the single *lacZ* gene, are sometimes, but not often found in lactobacilli such as in *L. bulgaricus* [12]. Lactobacilli have been studied intensively with respect to their enzymes for various different reasons, one of which is their ‘generally recognized as safe’ (GRAS) status and their safe use in food applications. It is anticipated that galacto-oligosaccharides (GOS) produced by these β-galactosidases will have better selectivity for growth and metabolic activity of this bacterial genus in the gut.

An economical, sustainable and intelligent use of biocatalysts can be achieved through immobilization, where the enzyme is bound onto a suitable food-grade carrier. Efforts have been made to immobilize β-galactosidases from *L. reuteri,* a LacLM-type, and *Lactobacillus bulgaricus,* a LacZ-type, on chitin using the chitin binding domain (ChBD) of *Bacillus circulans* WL-12 chitinase A1 [17]. Cell surface display has been shown as a new strategy for enzyme immobilization, which involves the use of food-grade organism *L. plantarum* both as a cell factory for the production of enzymes useful for food applications and as the carrier for the immobilization of the over-expressed enzyme by anchoring the enzyme on the cell surface [18,19]. This enables the direct use of the microbial cells straight after the fermentation step as an immobilized biocatalysts, offering the known advantages of immobilization (reuse of enzyme, stabilization, etc.) together with a significant simplification of the production process since costly downstream processing of the cells producing the enzyme (cell disruption, protein purification, etc.) as well as the use of carrier material will not be necessary. We recently reported cell surface display of mannanolytic and chitinolytic enzymes in *L. plantarum* using two anchors from *L. plantarum,* a lipoprotein-anchor derived from the Lp_1261 protein and a cell wall anchor (cwa2) derived from the Lp_2578 protein [19]. However, this approach works less efficient with dimeric and oligomeric enzymes, such as β-galactosidases from lactobacilli, due to low secretion efficiency of target proteins. Therefore, it is of our interest to find another strategy to display lactobacillal β-galactosidases on *Lactobacillus* cell surface for use as immobilized biocatalysts for applications in lactose conversion and GOS formation processes.

There are two principally different ways of anchoring a secreted protein to the bacterial cell wall: covalently, via the sortase pathway, or non-covalently, via a protein domain that interacts strongly with cell wall components. In sortase-mediated anchoring, the secreted protein carries a C-terminal anchor containing the so-called LP × TG motif followed by a hydrophobic domain and a positively charged tail [20]. The hydrophobic domain and the charged tail keep the protein from being released to the medium, thereby allowing recognition of the LP × TG motif by a membrane-associated transpeptidase called sortase [20–22]. The sortase cleaves the peptide bond between threonine and glycine in the LP X TG motif and links the now C-terminal threonine of the surface protein to a pentaglycine in the cell wall [21–25]. One of the non-covalent cell display systems exploits so-called LysM domains, the peptidoglycan binding motifs, that are known to promote cell wall association of several natural proteins [23,26]. These domains have been used to display proteins in lactic acid bacteria (LAB) by fusing the LysM domain N- or C-terminally to the target protein [27–30]. In *L. plantarum* WCFS1 ten proteins are predicted to be displayed at the cell wall through LysM domains [31].

In this present study, we exploit a single LysM domain derived from the Lp_3014 protein in *L. plantarum* WCFS1 for external attachment of two lactobacillal β-galactosidases, a LacLM-type from *L. reuteri* and a LacZ-type from *L. bulgaricus,* on the cell surface of four *Lactobacillus* species. The immobilization of active β-galactosidases through cell-surface display can be utilized as safe and stable non-GMO food-grade biocatalysts that can be used in the production of prebiotic GOS.

## 2 Results

### 2.1 Expression of Recombinant *Lactobacillal* β-galactosidases in *E. coli*

**Figure 1:**
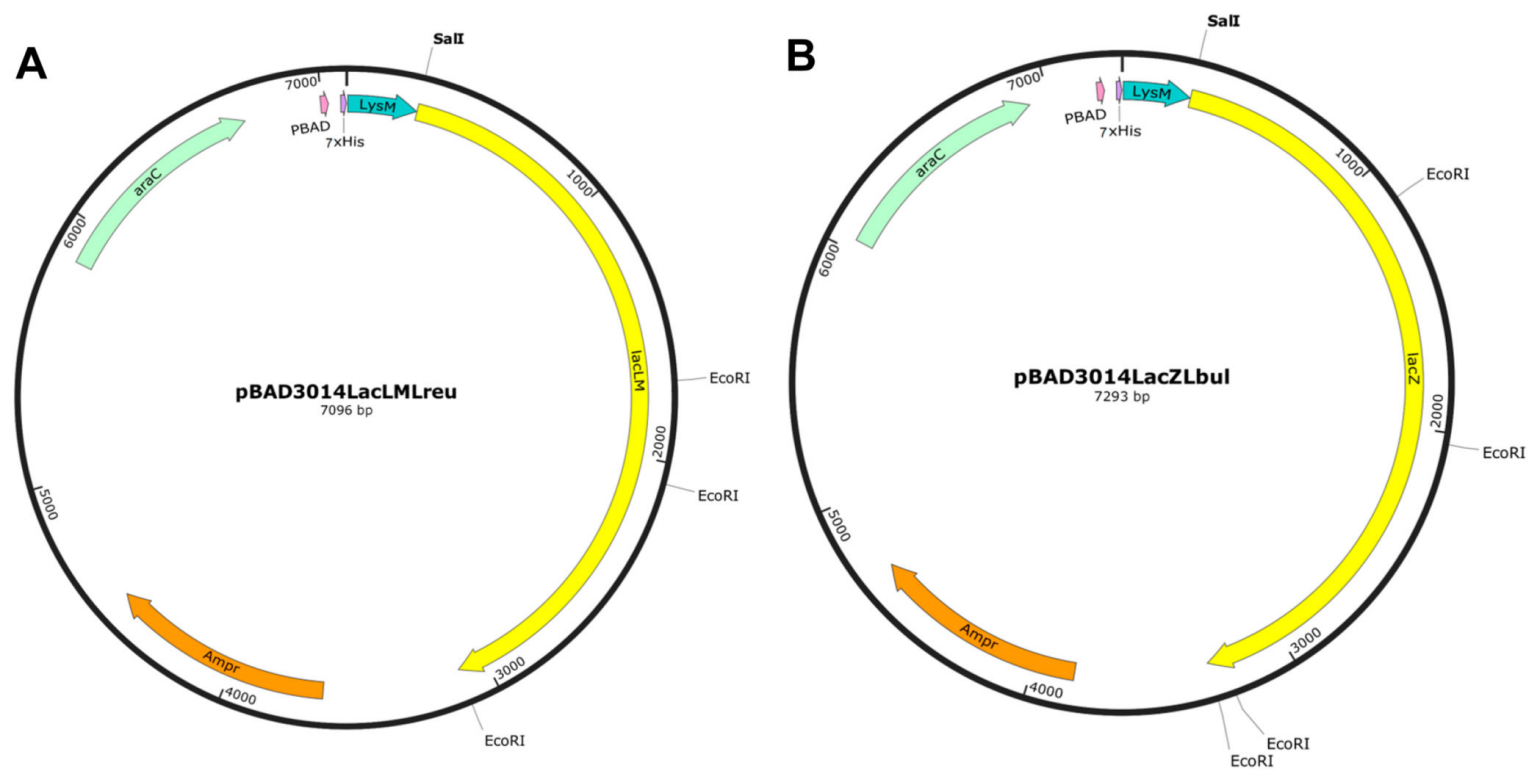
The expression vectors for LysM-LacLMLreu (**A**) and LysM-LacZLbul (**B**) in *E. coli*. The vectors are the derivatives of the pBAD vector (Invitrogen, Carlsbad, CA, USA) containing a 7 × His tag sequence fused to a single LysM domain from Lp_3014, *L. plantarum* WCFS1. LacLMLreu encoded by two overlapping genes *lacLM* and LacZLbul encoded by the *lacZ* gene are the β-galactosidases from *L. reuteri* and *L. delbrueckii* subsp. *bulgaricus* DSM 20081, respectively. See text for more details.

The overlapping *lacLM* genes from *L. reuteri* L103 and the *lacZ* gene from *L. bulgaricus* DSM20081, both encoding β-galactosidases, were fused N-terminally to the LysM motif for expression and later attachment of the hybrid proteins to the peptidoglycan layer of *Lactobacillus* spp. An 88 residue fragment of the LysM motif from the 204-residue-Lp_3014 protein of an extracellular transglycosylase of *L. plantarum* WCFS1 [31,32] was fused to two β-galactosidases for production in *E. coli*. The two hybrid sequences were then cloned into the expression vector pBAD containing an N-terminal 7 × Histidine tag for immunodetection, yielding pBAD3014LacLMLreu and pBAD3014LacZLbul (Figure 1).

**Figure 2:**
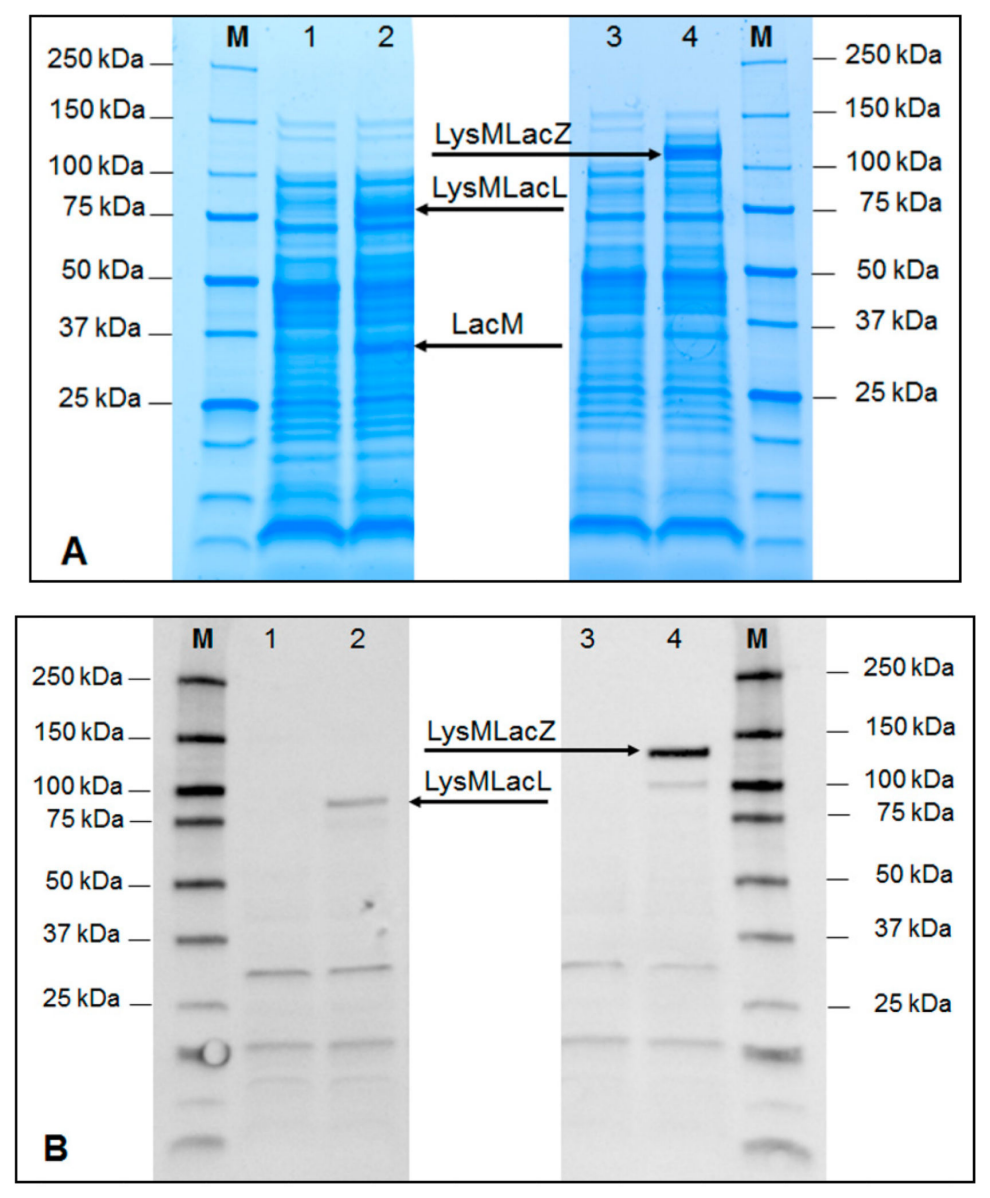
SDS-PAGE analysis (**A**) and Western blot analysis (**B**) of a cell-free extract of crude β-galactosidase fusion proteins, LysM-LacLMLreu (non-induced: lane 1, induced: lane 2) and LysM-LacZLbul (non-induced: lane 3, induced: lane 4), overexpressed in *E. coli* HST08. LacLMLreu encoded by two overlapping genes *lacLM* and LacZLbul encoded by *lacZ* gene are the β-galactosidases from *L. reuteri* and *L. delbrueckii* subsp. *bulgaricus* DSM 20081, respectively. The cultivation and induction conditions are as described in Materials and Methods and samples were taken at different time points after induction during cultivations. The arrows indicate the subunits of the recombinant β-galactosidases. M denotes the Precision protein ladder (Biorad, CA, USA).

The *E. coli* strains were cultivated in Luria-Bertani (LB) medium, induced for gene expression (as described in Materials and Methods), and the SDS-PAGE and Western blot analyses of cell-free extracts (Figure 2) showed the production of the two recombinant β-galactosidases, LysM-LacLMLreu and LysM-LacZLbul. As judged by SDS-PAGE (Figure 2A), LysM-LacLMLreu shows two bands with apparent molecular masses corresponding to a large subunit (LacL) and a small subunit (LacM) at ~90 kDa and ~35 kDa. These values are in agreement with reported molecular masses of 73 and 35 kDa for these two subunits of β-galactosidase from *L. reutei* [5,16]. The increase in molecular mass of a larger subunit in LysM-LacLMLreu is due to the added His-LysM fragment (~18 kDa). On the other hand, β-galactosidase from *L. bulgaricus* was reported to be a homodimer, consisting of two identical subunits of ~115 kDa [12]. A unique band of ~130 kDa corresponding to the molecular mass of a single subunit of LacZ fused with the 18 kDa-fragment of the histidine-tag and the LysM domain was shown on SDS-PAGE analysis of a cell-free extract of LysM-LacZLbul as expected (Figure 2A). Western blot analysis of the crude, cell-free extracts was performed using anti-His antibody for detection. Figure 2B shows that the recombinant bacteria produced the expected proteins, LysMLacL (lane 2) and LysMLacZ (lane 4). LacM was not detected as it does not contain the histidine-tag.

To check if the heterologously produced enzymes were functionally active, β-galactosidase activities of cell-free lysates of *E. coli* cells carrying different expression vectors were measured. The highest yields obtained for the two recombinant enzymes were 11.1 ± 1.6 k·U_*o*NPG_ per L of medium with a specific activity of 6.04 ± 0. 03 U·mg^−1^ for LysM-LacLMLreu and 46.9 ± 2.7 kU_*o*NPG_ per L of medium with a specific activity of 41.1 ± 0.9 U·mg^−1^ for LysM-LacZLbul, respectively (Table 1). The β-galactosidase activities in non-induced *E. coli* cells were negligible for both LysM-LacLMLreu and LysM-LacZLbul showing that the activity is from the overproduced β-galactosidases (Table 1).

### 2.2 Display of Lactobacillal β-Galactosidases on Lactobacillus Cell Surface

**Figure 3:**
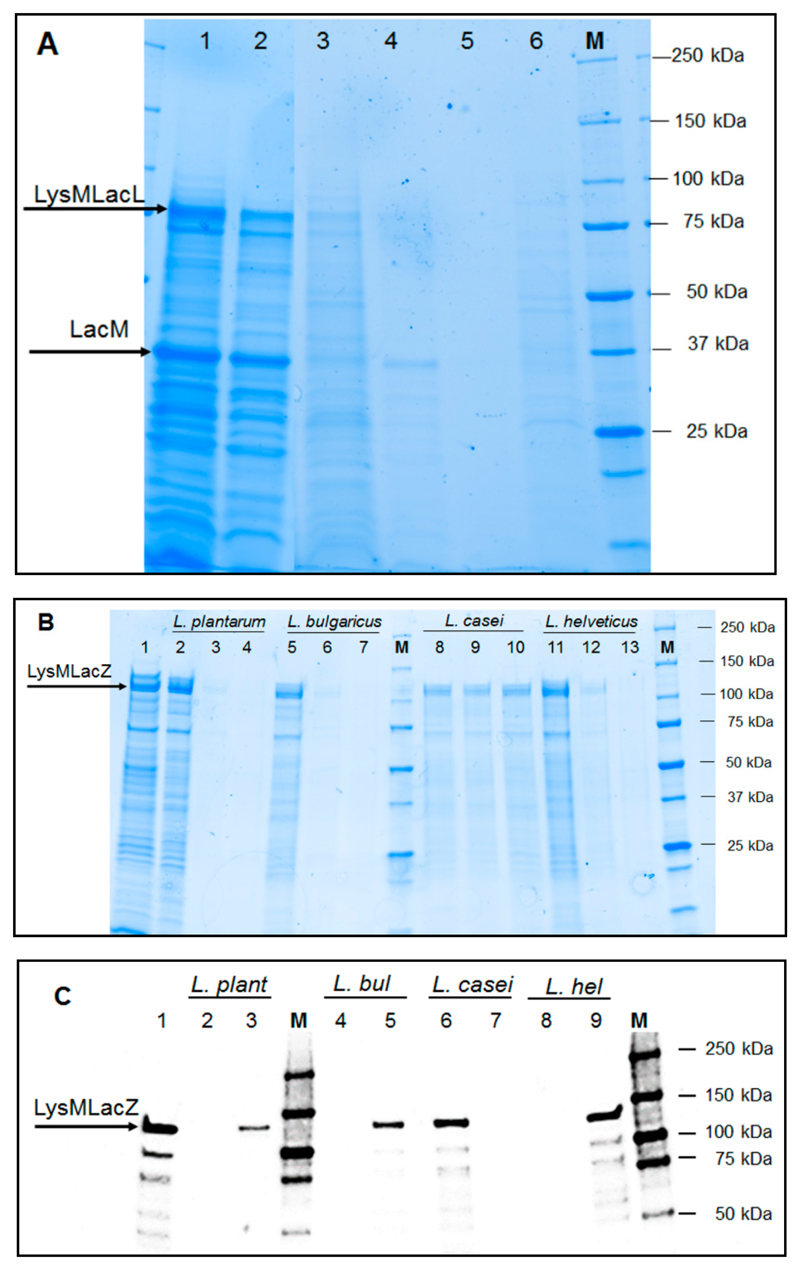
SDS-PAGE analysis (**A,B**) and Western blot analysis **(C)** of immobilization of recombinant enzymes. LacLMLreu encoded by two overlapping genes *lacLM* and LacZLbul encoded by *lacZ* gene are the β-galactosidases from *L. reuteri* and *L. delbrueckii* subsp. *bulgaricus* DSM 20081, respectively. The arrows indicate the subunits of the recombinant β-galactosidases. M denotes the Precision protein ladder (Biorad, CA, USA). (**A**) Cell-free crude extracts of *E. coli* HST08 harboring pBAD3014LacLMLreu (containing LysM-LacLMLreu) at 18 h of induction (lane1); flow through during immobilization (lane 2); surface anchored-LysM-LacLMLreu in *L. plantarum* WCFS1 (lane 3) and washing fractions (lanes 4, 5); non-displaying *L. plantarum* WCFS1 cells, negative control (lane 6). (**B**) Cell-free crude extracts of *E. coli* HST08 harboring pBAD3014LacZLbul (containing LysM-LacZLbul) at 18 h of induction (lane1); flow through during immobilization on the cell surface of *L. plantarum* WCFS1 (lane 2) and washing fractions (lanes 3, 4); flow through during immobilization on the cell surface of *L. delbrueckii* subsp. *bulgaricus* DSM 20081 (lane 5) and washing fractions (lanes 6, 7); flow through during immobilization on cell surface of *L. casei* (lane 8) and washing fractions (lanes 9, 10); flow through during immobilization on cell surface of *L. helveticus* DSM 20075 (lane 11) and washing fractions (lanes 12, 13). (**C**) Cell-free crude extracts of *E. coli* HST08 harboring pBAD3014LacZLbul (containing LysM-LacZLbul) at 18 h of induction (lane 1); non-displaying *L. plantarum* WCFS1 cells (lane 2) and surface anchored-LysM-LacZLbul in *L. plantarum* WCFS1 (lane 3); non-displaying *L. delbrueckii* subsp. *bulgaricus* DSM 20081 cells (lane 4) and surface anchored-LysM-LacZLbul in *L. delbrueckii* subsp. *bulgaricus* DSM 20081 (lane 5); surface anchored-LysM-LacZLbul in *L. casei* (lane 6) and non-displaying *L. casei* cells (lane 7); non-displaying *L. helveticus* DSM 20075 cells (lane 8) and surface anchored-LysM-LacZLbul in *L. helveticus* DSM 20075 (lane 9).

**Figure 4:**
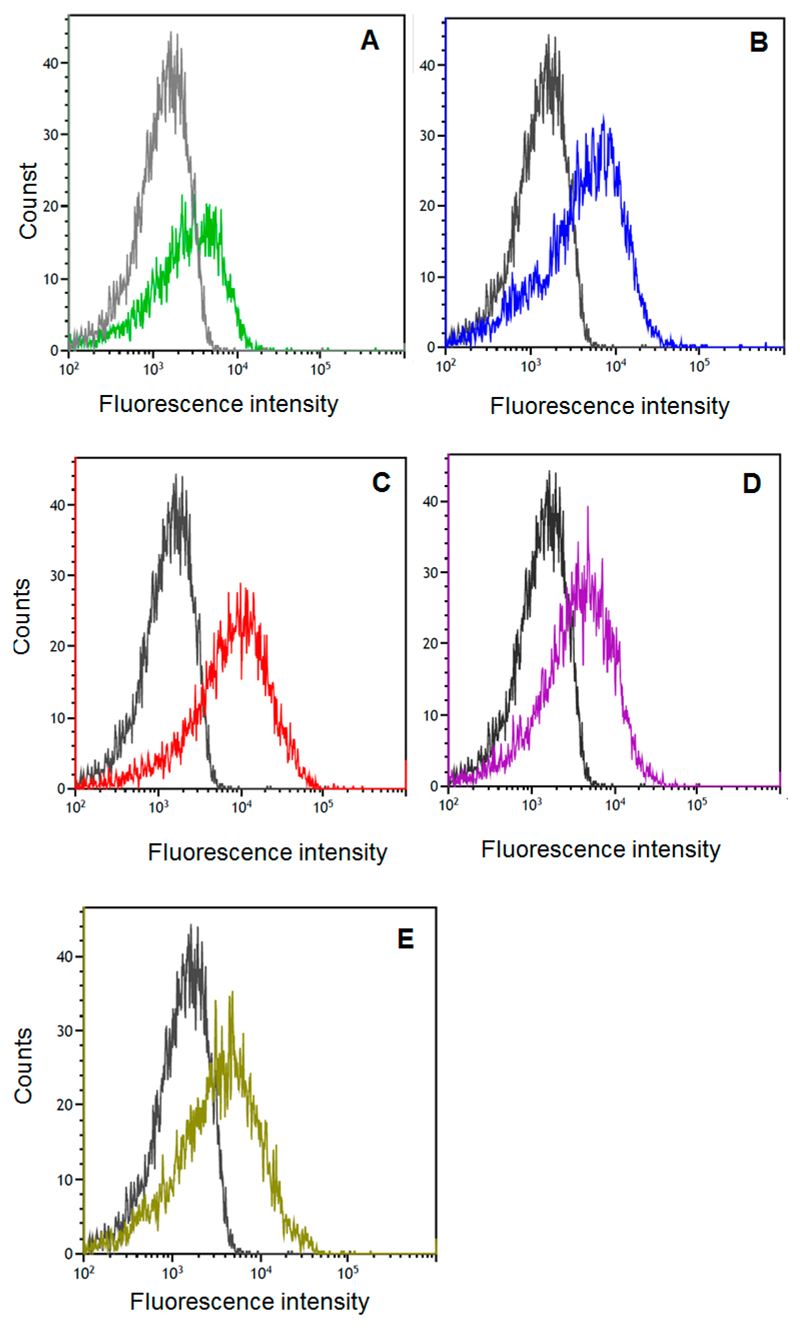
Analysis of surface localization of LysM-LacLMLreu and LysM-LacZLbul in *Lactobacillus* cells by using flow cytometry: surface anchored-LysM-LacLMLreu in *L. plantarum* WCFS1 (**A**, green line); surface anchored-LysM-LacZLbul in *L. plantarum* WCFS1 (**B**, blue line), in *L. delbrueckii* subsp. *bulgaricus* DSM 20081 (**C**, red line), in *L. casei* (**D**, purple line) and in *L. helveticus* DSM 20075 (**E**, olive line). Non-displaying *Lactobacillus* cells were used as negative controls (A–E, black line).

To investigate the attachment of the two hybrid proteins, LysM-LacLMLreu and LysM-LacZLbul, to the cell wall of *L. plantarum*, cell-free crude extracts from *E. coli* harboring β-galactosidases corresponding to 50 U_*o*NPG_ (~5–6 mg protein) were incubated with *L. plantarum* cells collected from one mL cultures at OD_600_ ~4.0. The enzymes and *L. plantarum* were incubated at 37 °C with gentle agitation, and after 24 h of incubation, the residual activities in the supernatant as well as on the cell surface were determined for both enzymes (Table 2A). The immobilization yield (IY) is a measure of how much of the applied protein bound to the surface of *Lactobacillus* cells. Immobilizations yields for LysM-LacLMLreu and LysM-LacZLbul were 6.5% and 31.9%, respectively. SDS-PAGE analysis of the samples after the immobilization procedure showed strong bands of LysM-LacL and LacM or LysM-LacZ in the residual supernatants (Figure 3A, lane 2; Figure 3B, lane 2), indicating relatively high amounts of non-anchored proteins in the supernatants. Two successive washing steps with 50 mM sodium phosphate buffer (NaPB, pH 6.5) did not release the enzymes showing that the immobilization is both effective and stable (Figure 3A, lanes 4, 5; Figure 3B, lanes 3, 4). The low immobilization yield for LysM-LacLMLreu was confirmed by the SDS-PAGE analysis (Figure 3A, lane 3) Western blot analysis of the crude, cell-free extracts of *L. plantarum* LacZLbul-displaying cells was performed using an anti-His antibody for detection showing the presence of LacZLbul (Figure 3C; lane 3). Flow cytometry confirmed the surface localization of both enzymes LysM-LacLMLreu and LysM-LacZLbul as clear shifts in the fluorescence signals for *L. plantarum* LacLMLreu- and LacZLbul-displaying cells in comparison to the control strain were observed (Figure 4A,B). The surface-displayed enzymes were shown to be functionally active. β-Galactosidase activities obtained for *L. plantarum* displaying cells were 179 ant 1153 U per g dry cell weight, corresponding to approximately 0.99 and 4.61 mg of active, surface-anchored β-galactosidase per g dry cell mass for LysM-LacLMLreu and LysM-LacZLbul (Table 2A), respectivel.

Due to higher immobilization yields and increased amounts of active surface-anchored protein in *L. plantarum,* LysM-LacZLbul was chosen for further analysis of its display on the cell surface of other food-relevant *Lactobacillus* spp. including *L. bulgaricus, L. casei* and *L. helveticus*. The parameters of residual activities in the supernatant after the anchoring experiment, activity on the cell surface, immobilization yields, activity retention and amounts of active surface-anchored LysM-LacZLbul were determined and are presented in Table 2B. It was shown that surface-anchored LysM-LacZLbul was released from the cell surface of *L. casei* during the subsequent washing steps (Figure 3B, lanes 9, 10). Western blot analysis of the crude, cell-free extracts of *Lactobacillus* LysM-LacZLbul-displaying cells indicated the binding of LysM-LacZLbul to all four *Lactobacillus* spp. tested (Figure 3C; lanes 3, 5, 6, 9) as was also confirmed by flow cytometry (Figure 4B–E). *L. plantarum* bound most efficiently among the tested *Lactobacillus* species shown by the highest immobilization yield and the highest amount of active, surface-anchored LysM-LacZLbul (Table 2B).

### 2.3 Enzymatic Stability of β-Galactosidase-Displaying Cells

Both temperature stability and reusability of β-galactosidase displaying cells were determined. For temperature stability, *L. plantarum* galactosidase-displaying cells were incubated in 50 mM sodium phosphate buffer (NaPB), pH 6.5 at different temperatures, and at certain time intervals, the residual β-galactosidase activities on *L. plantarum* cell surface were measured. Both LysM-LacLMLreu and LysM-LacZLbul-displaying cells are very stable at –20 °C with a half-life time of activity $\left({\text{τ}}_{\frac{1}{2}}\right)$ of approximately 6 months (Table 3). The half-life time of activity of LysM-LacLMLreu-displaying cells at 30 °C is 55 h, whereas half-life times of activity of LysM-LacZLbul-displaying cells at 30 °C and 50 °C are 120 h and 30 h, respectively (Table 3).

**Figure 5:**
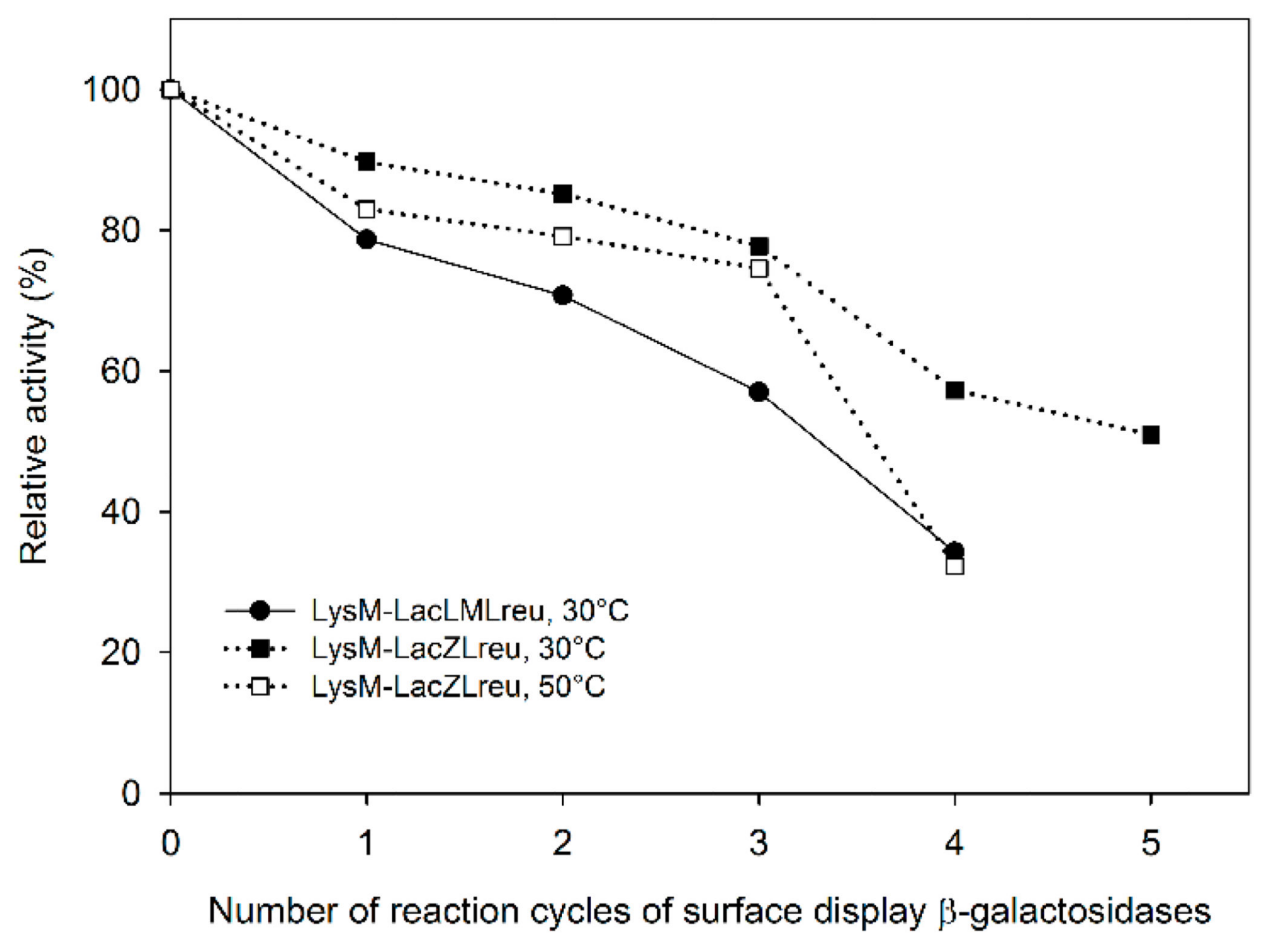
Enzymatic activity of surface display β-galactosidases, LysM-LacLMLreu- and LysM-LacZLbul, during several repeated rounds of lactose conversion using *L. plantarum* WCFS1 displaying cells. Experiments were performed in duplicates, and the standard deviation was always less than 5%.

To test the reusability of LysM-LacLMLreu- and LysM-LacZLbul-displaying cells, the enzyme activity was measured during several repeated rounds of lactose conversion with two washing steps between each cycle. The enzymatic activities of *L. plantarum* LysM-LacZLbul-displaying cells decreased by ~23% and 27% at 30 °C and 50 °C (Figure 5), respectively, after three conversion/washing cycles, indicating that these displaying cells can be reused for several rounds of biocatalysis at tested temperatures. LysM-LacLMLreu-displaying cells are less stable than LysM-LacZLbul-displaying cells as only 56% of the initial β-galactosidase activity are retained at 30 °C after the third cycle (Figure 5). LysM-LacZLbul-displaying cells retained 35% of β-galactosidase activity after the fourth cycle at 50 °C, 57% and 51% after the fourth and fifth cycle, respectively, at 30 °C (Figure 5). These observations indicate that immobilized fusion LysM-β-galactosidases can be reused for at least four to five repeated rounds of lactose conversion.

### 2.4 Formation of Galacto-Oligosaccharides (GOS)

**Figure 6:**
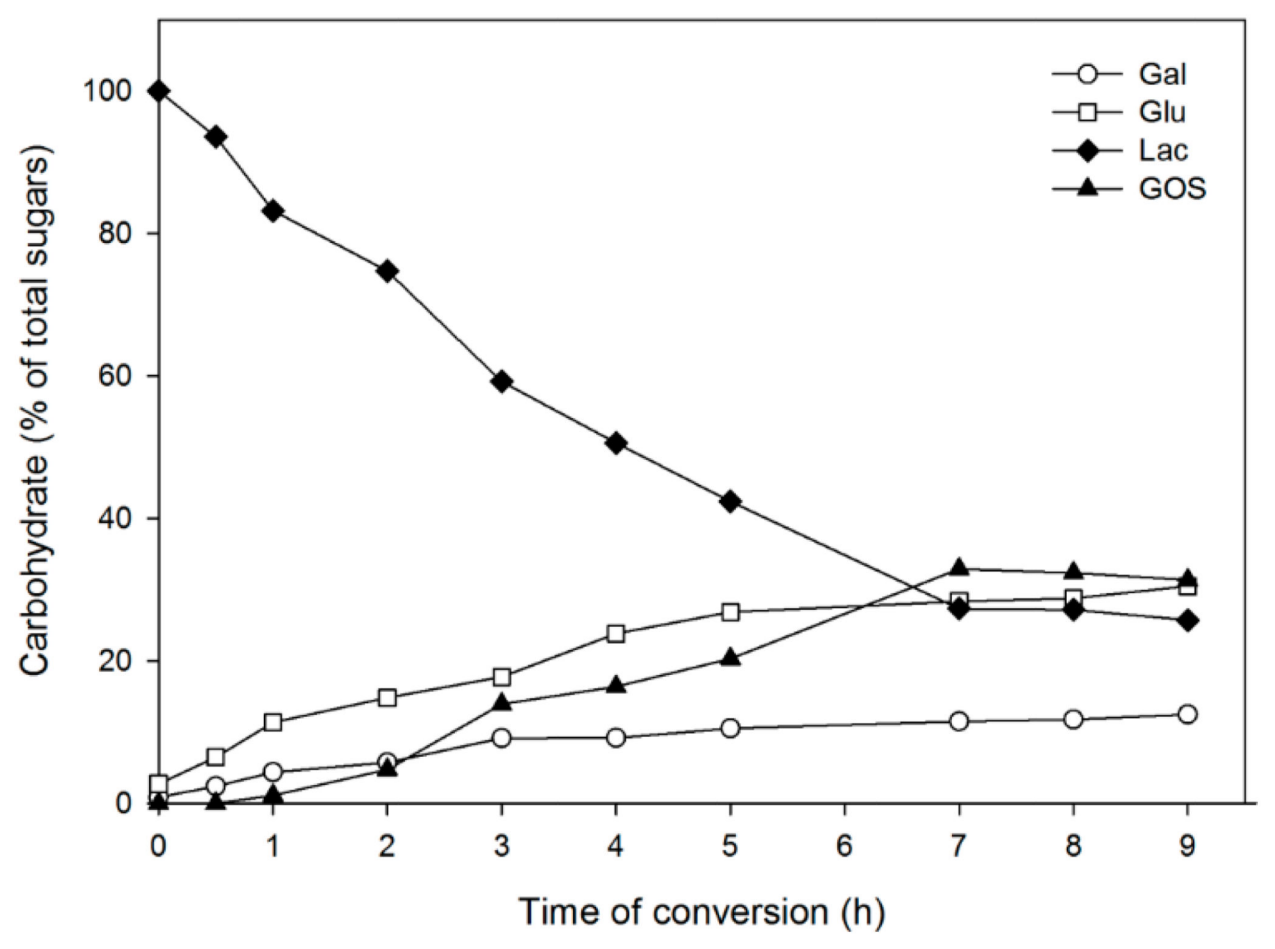
Course of reaction for lactose conversion by surface display β-galactosidase from L. *bulgaricus* (LysM-LacZLbul) in *L. plantarum* WCFS1 as determined by HPLC. The batch conversion was carried out at 30 °C using 205 g/L initial lactose concentration in 50 mM NaPB (pH 6.5) and constant agitation (500 rpm). *L. plantarum* LysM-LacZLbul displaying cells were added to equivalent concentrations of 1.0 U_Lac_/mL of the reaction mixture. Experiments were performed in duplicates, and the standard deviation was always less than 5%.

Figure 6 shows the formation of GOS using *L. plantarum* cells displaying β-galactosidase LacZ from *L. bulgaricus* (LysM-LacZLbul) with 1.0 U_Lac_ β-galactosidase activity per mL of the reaction mixture and 205 g/L initial lactose in 50 mM sodium phosphate buffer (pH 6.5) at 30°C. The maximal GOS yield was around 32% of total sugars obtained at 72% lactose conversion after 7 h of conversion. This observation shows that surface-displayed LacZ is able to convert lactose to form galacto-oligosaccharides. We could identify the main GOS products of transgalactosylation, which are β-D-Gal*p*-(1→6)-D-Glc, β-D-Gal*p*-(1→3)-D-Lac, β-D-Gal*p*-(1→3)-D-Glc, β-D-Gal*p*-(1→3)-D-Gal, β-D-Gal*p*-(l→6)-D-Gal, and β-D-Gal*p*-(1→6)-D-Lac. This is similar to the product profile when performing the conversion reaction with the free enzyme as previously reported [12].

## 3 Discussion

Surface display of proteins on cells of lactic acid bacteria (LAB) generally requires genetic modifications, which might have limitations in food and medical applications due to the sensitive issue of the use of genetically modified organisms (GMO). Anchoring heterologous proteins on the cell surface of non-genetically modified LAB (non-GMO) via mediated cell wall binding domains including surface layer domain (SLPs) [33,34], LysM domain [26,30,35–37], W × L domains [38] attracts increasing interest.

Lysin motif (LysM) domains are found in many bacterial peptidoglycan hydrolases [26,38,39]. Peptidoglycan contains sugar (glycan) chains, which consist of *N*-acetylglucosamine (NAG) and *N*-acetylmuramic acid (NAM) units joined by glycosidic linkages. Proteins harboring LysM motifs have been shown to bind non-covalently to the peptidoglycan layer and have been employed to display heterologous proteins on the bacterial cell-surface [26,40,41]. These domain can contain single or multiple LysM motifs [41], and they have been used to display proteins in LAB by fusion either to the N- or C-terminus of a target protein [27–30]. Interestingly, the LysM motif derived from the *L. plantarum* Lp_3014 transglycosylase has been used successfully for surface display of invasin [36] and a chemokine fused to an HIV antigen [37] previously.

In this work, we used the single LysM domain derived from Lp_3014 to anchor two different lactobacillal β-galactosidases, a heterodimeric type from *L. reuteri* and a homodimeric type from *L. bulgaricus,* on the cell surface of four species of lactobacilli. Functional active fusion proteins, LysM-LacLMLreu and LysM-LacZLbul, were successfully expressed in *E. coli*. However, the expression yield of LysM-LacLMLreu was ten-fold lower than that of the β-galactosidase from *L. reuteri* (LacLMLreu) without LysM expressed previously in *E. coli*, which was reported to be 110 kU of β-galactosidase activity per liter of cultivation medium [16]. This may indicate that the fusion of the LysM domain has a negative effect on the expression level. Interestingly, the expression yields of LysM-LacZLbul were 4-fold and 7-fold higher in terms of volumetric and specific activities, respectively. than that of LysM-LacLMLreu using the same host, expression system and induction conditions. β-Galactosidase from *L. bulgaricus* (LysM-LacZLbul) is a homodimer whereas β-galactosidase from *L. reuteri* (LysM-LacLMLreu) is a heterodimer, and hence the fusion of the LysM domain only to the LacL subunits might lead to the discrepancy between the yields of these two fusion proteins due to different folding mechanisms.

Not surprisingly, the affinity for peptidoglycan of homodimeric LysM-LacZLbul is significantly higher than LysM-LacLMLreu as shown by the immobilization yield (Table 2A). As aforementioned LacLMLreu from *L. reuteri* is a heterodimer and the LysM domain is fused N-terminally to only LacL, while LacZLbul from *L. bulgaricus* is a homodimer, hence each of the identical subunits will carry its own LysM domain leading to stronger attachment of LacZ on the *L. plantarum* cell wall. This could be a likely explanation for the higher immobilization yields observed for LysM-LacZLbul. Even though the immobilization yields obtained in this study were significantly lower than the immobilization yields for these same enzymes when a chitin binding domain (ChBD) together with chitin was used [17], the activity retention (AR) on the *L. plantarum* cell surface (46.9% and 63.5% for LysM-LacLMLreu and LysM-LacZLbul, respectively) were significantly higher. The AR values for ChBD-LacLM, LacLM-ChBD and LacZ-ChBD using chitin beads were 19%, 26% and 13%, respectively [17]. Notably, the amount of active surface anchored LysM-LacLMLreu (0.99 ± 0.02 mg per g dry cell weight) on the cell surface of *L. plantarum* WCFS1 is significantly lower than LysM-LacZLbul (4.61 ± 0.05 mg per g dry cell weight). This is mainly due to the low immobilization yield of LysM-LacLMLreu. *L. plantarum* collected from one mL cultures at OD_600_ ~4.0 was used in immobilization reactions, hence the amount of *L. plantarum* cells was estimated to be ~3.0 × 10^9^ cfu/mL. Therefore, we calculated that 8.22 μg LysM-LacLMLreu and 38.3 μg LysM-LacZLbul anchored on 3.0 × 10^9^ *L. plantarum* cells or 0.002 pg LysM-LacLMLreu and 0.012 pg LysM-LacZLbul per *L. plantarum* cell. Xu et al. (2011) reported the use of the putative muropeptidase MurO (Lp_2162) from *L. plantarum* containing two putative LysM repeat regions for displaying a green fluorescent protein (GFP) and a β-galactosidase from *Bifidobacterium bifidum* on the surface of *L. plantarum* cells [42]. They reported that 0.008 pg of GFP was displayed per cell on non-treated *L. plantarum* cells, while the amount of active surface anchored β-galactosidase from *B. bifidum* on the surface of *L. plantarum* cells was not reported in that study.

Further, we tested the capability of binding the fusion protein LysM-LacZLbul to the cell wall of three other *Lactobacillus* species. *L. plantarum* showed the best capacity among the tested *Lactobacillus* for surface anchoring of LysM-LacZLbul (Table 2B), whereas *L. bulgaricus, L. casei* and *L. helveticus* are comparable in term of the amount of active surface-anchored enzyme.

The highest GOS yield of 32% obtained with the surface-immobilized enzyme is lower than the yield obtained with the free enzyme LacZ from *L. bulgaricus* (Figure 6), which was previously reported to be approximately 50% [12]. This could be due to the binding of LysM-LacZLbul to the peptidoglycan and the attachment of the enzyme on *Lactobacillus* cell surface, which might hinder the access of the substrate lactose to the active site of the enzyme. Interestingly, the GOS yield obtained from lactose conversion using *L. plantarum* cells displaying β-galactosidase (LysM-LacZLbul) from *L. bulgaricus* is significantly higher than the yield obtained with immobilized β-galactosidase (LacZ-ChBD) on chitin, which was previously reported around 23%–24% [12]. It indicates that β-galactosidase from *L. bulgaricus* anchored on *L. plantarum* cell surface is more catalytically efficient than its immobilized form on chitin.

## 4 Materials and Methods

### 4.1 Bacterial Strains and Culture Conditions

The bacterial strains and plasmids used in this study are listed in Table 4. *Lactobacillus plantarum* WCFS1, isolated from human saliva as described by Kleerebezem et al. [32], was originally obtained from NIZO Food Research (Ede, The Netherlands) and maintained in the culture collection of the Norwegian University of Life Sciences, Ås, Norway. *L. helveticus* DSM 20075 (ATCC 15009) and *L. delbrueckii* subsp. *bulgaricus* DSM 20081 (ATCC 11842) were obtained from the German Collection of Microorganisms and Cell Cultures (DSMZ; Braunschweig, Germany). *L. casei* was obtained from the culture collection of the Food Biotechnology Laboratory, BOKU-University of Natural Resources and Life Sciences Vienna. *Lactobacillus* strains were cultivated on MRS medium (*Lactobacillus* broth according to De Man, Rogosa and Shape [43]) (Carl Roth, Karlsruhe, Germany) at 37 °C without agitation. *E. coli* NEB5α (New England Biolabs, Frankfurt am Main, Germany) was used as cloning hosts in the transformation of DNA fragments; whereas *E. coli* HST08 (Clontech, Mountain View, CA, USA) was used as the expression host strain. *E. coli* strains were cultivated in Luria-Bertani (LB) medium (10g/L tryptone, 10 g/L NaCl, and 5 g/L yeast extract) at 37 °C with shaking at 140 rpm. Agar media were prepared by adding 1.5% agar to the respective media. When needed, ampicillin was supplemented to media to a final concentration of 100 μg/mL for *E. coli* cultivations.

### 4.2 Chemicals, Enzymes and Plasmids

All chemicals and enzymes were purchased from Sigma (St. Louis, MO, USA) unless stated otherwise and were of the highest quality available. All restriction enzymes, Phusion high-fidelity DNA polymerase, T4 DNA ligase, and corresponding buffers were from New England Biolabs (Frankfurt am Main, Germany). Staining dyes, DNA and protein standard ladders were from Bio-Rad (Hercules, CA, USA). All plasmids used in this study are listed in Table 4.

### 4.3 DNA Manipulation

Plasmids were isolated from *E. coli* strains using Monarch Plasmid Miniprep Kit (New England Biolabs, Frankfurt am Main, Germany) according to the manufacturer’s instructions. PCR amplifications of DNA were done using Q5 High-Fidelity 2X Master Mix (New England Biolabs). The primers used in this study, which were supplied by VBC-Biotech Service (Vienna, Austria), are listed in Table 5. PCR products and DNA fragments obtained by digestion with restriction enzymes were purified using Monarch DNA Gel Extraction Kit (New England Biolabs); and the DNA amounts were estimated using Nanodrop 2000 (Thermo Fisher Scientific, Waltham, MA, USA). The sequences of PCR-generated fragments were verified by DNA sequencing performed by a commercial provider (Microsynth, Vienna, Austria). The ligation of DNA fragments was performed using NEBuilder HiFi Assembly Cloning Kit (New England Biolabs). All plasmids were transformed into *E. coli* NEB5α chemical competent cells following the manufacturer’s protocol for obtaining the plasmids in sufficient amounts. The constructed plasmids (Table 4) were chemically transformed into expression host strain *E. coli* HST08.

### 4.4 Plasmid Construction

Two recombinant fusion proteins were constructed. The first fusion protein was based on LacLM from *L. reuteri* and the LysM domain attached upstream of LacLM (termed LysM-LacLMLreu). The second fusion protein was based on LacZ from *L. delbrueckii* subsp. *bulgaricus* DSM 20081 and the LysM domain attached upstream of LacZ (termed LysM-LacZLbul). Plasmid pBAD_3014AgESAT_DC (Table 4) [44] (was used for the construction of the expression plasmids. This plasmid is a derivate of pBAD vector (Invitrogen, Carlsbad, CA, USA) containing a 7 × His tag sequence and a single LysM domain from Lp_3014, which is a putative extracellular transglycosylase with LysM peptidoglycan binding domain from *L. plantarum* WCFS1 (NCBI reference sequence no. NC_004567.2) [31,32], fused to the hybrid tuberculosis antigen AgESAT-DC [44]. The fragment of *lacLM* genes from *L. reuteri* was amplified from the plasmid pHA1032 (Table 4) [16] with the primer pair Fwd1LreuSalI and Rev1LreuEcoRI (Table 5), whereas the *lacZ* gene from *L. bulgaricus* was amplified from the plasmid pTH103 (Table 4) [12] with the primer pair Fwd2LbulSalI and Rev2LbulEcoRI (Table 5). The PCR-generated products were then cloned into *Sal*I and *EcoR*I cloning sites of the pBAD_3014AgESAT_DC vector using and NEBuilder HiFi DNA Assembly Cloning Kit (New England Biolabs) following the manufacturer’s instructions, resulting in two expression plasmids pBAD3014LacLMLreu and pBAD3014LacZLbul (Figure 1).

### 4.5 Gene Expression in *E. coli*

The constructed plasmids pBAD3014LacLMLreu and pBAD3014LacZLbul were chemically transformed into expression host *E. coli* HST08. For gene expression, overnight cultures of *E. coli* HST08 were diluted in 300 mL of fresh LB broth containing 100 μg/mL ampicillin to an OD_600_ of ~0.1 and incubated at 37 °C with shaking at 140 rpm to an OD_600_ ~0.6. Gene expression was then induced by L-arabinose to a final concentration of 0.7 mg/mL and the cultures were incubated further at 25 °C for 18 h with shaking at 140 rpm. Cells were harvested at an OD_600_ of ~3.0 by centrifugation at 4000× *g* for 30 min at 4 °C, washed twice, and resuspended in 50 mM sodium phosphate buffer (NaPB), pH 6.5. Cells were disrupted by using a French press (AMINCO, Maryland, USA). Debris was removed by centrifugation (10,000× *g* for 15 min at 4 °C) to obtain the crude extract.

### 4.6 Immobilization of β-Galactosidases on Lactobacillus Cell Surface

One mL of *Lactobacillus* cultures were collected at OD_600_ ~4.0 by centrifugation (4000× *g* for 5 min at 4 °C) and the cells were washed with 50 mM sodium phosphate buffer (NaPB), pH 6.5. The cell pellets were then mixed with one mL of diluted cell-free crude extracts of 50 U_*o*NPG_/mL (~5–6 mg protein/mL) of fused LysM-β-galactosidases (LysM-LacLMLreu or LysM-LacZLbul) and incubated at 37 °C for 24 h with gentle agitation. *Lactobacillus* β-galactosidase displaying cells were separated from the supernatants by centrifugation (4000× *g* for 5 min at 4 °C). Cells were then washed with NaPB (pH 6.5) two times; the supernatants and wash solutions were collected for SDS-PAGE analysis and activity and protein measurements. *Lactobacillus* β-galactosidase displaying cells were resuspended in NaPB (pH 6.5) for further studies.

### 4.7 Protein Determination

Protein concentrations were determined using the method of Bradford [45] with bovine serum albumin (BSA) as standard.

### 4.8 β-Galactosidase Assays

β-Galactosidase activity was determined using *o*-nitrophenyl-β-D-galactopyranoside (*o*NPG) or lactose as the substrates as previously described [5] with modifications. When chromogenic substrate *o*NPG was used, the reaction was started by adding 20 μL of *Lactobacillus* β-galactosidase displaying cell suspension to 480 μL of 22 mM *o*NPG in 50 mM NaBP (pH 6.5) and stopped by adding 750 μL of 0.4 M Na_2_CO_3_ after 10 min of incubation at 30 °C. The release of o-nitrophenol (*o*NP) was measured by determining the absorbance at 420 nm. One unit of *o*NPG activity was defined as the amount of β-galactosidase releasing 1 μmol of *o*NP per minute under the defined conditions.

When lactose was used as the substrate, 20 μL of *Lactobacillus* β-galactosidase displaying cell suspension was added to 480 μL of a 600 mM lactose solution in 50 mM sodium phosphate buffer, pH 6.5. After 10 min of incubation at 30 °C, the reaction was stopped by heating the reaction mixture at 99 °C for 5 min. The reaction mixture was cooled to room temperature, and the release of D-glucose was determined using the test kit from Megazyme. One unit of lactase activity was defined as the amount of enzyme releasing 1 μmol of D-glucose per minute under the given conditions.

### 4.9 Gel Electrophoresis Analysis

For visual observation of the expression level of the two recombinant β-galactosidases (LysM-LacLMLreu and LysM-LacZLbul) in *E. coli* and the effectiveness of the immobilization, cell-free extracts, supernatants, and wash solutions were analyzed by Sodium Dodecyl Sulfate Polyacrylamide Gel Electrophoresis (SDS-PAGE). Protein bands were visualized by staining with Bio-safe Coomassie (Bio-Rad). The determination of protein mass was carried out using Unstained Precision plus Protein Standard (Bio-Rad).

### 4.10 Western Blotting

Proteins in the cell-free extracts were separated by SDS-PAGE. Protein bands were then transferred to a nitrocellulose membrane using the Trans-Blot Turbo™ Transfer System (Biorad) following the manufacturer’s instructions. Monoclonal mouse anti-His antibody (Penta His Antibody, BSA-free) was obtained from Qiagen (Hilden, Germany), diluted 1:5000 and used as recommended by the manufacturer. The protein bands were visualized by using polyclonal rabbit anti-mouse antibody conjugated with horseradish peroxidase (HRP) (Dako, Denmark) and the Clarity™ Western ECL Blotting Substrate from Bio-Rad (Hercules, CA, USA).

### 4.11 Flow Cytometry

*Lactobacillus* β-galactosidase displaying cells were resuspended in 50 μL of phosphate buffered saline (PBS) (137 mM NaCl, 2.7 mM KCl, 2 mM KH_2_PO_4_, and 10 mM Na_2_HPO_4_, pH 7.4) containing 2% of BSA (PBS-B) and 0.1 μL of Penta His Antibody, BSA-free (Qiagen; diluted 1:500 in PBS-B). After incubation at RT for 40 min, the cells were centrifuged at 4000× *g* for 5 min at 4 °C and washed three times with 500 μL PBS-B. The cells were subsequently incubated with 50 μL PBS-B and 0.1 μL anti-mouse IgG H&L/Alexa Flour 488 conjugate (Cell Signaling Technology, Frankfurt am Main, Germany, diluted 1:750 in PBS-B) for 40 min in the dark at room temperature. After washing five times with 500 μL PBS-B, the stained cells were analyzed by flow cytometry using a CytoFLEX Flow Cytometer (Beckman Coulter, Brea, CA, USA) following the manufacturer’s instructions.

### 4.12 Temperature Stability and Reusability of Immobilized Enzymes

The temperature stability of immobilized enzymes was studied by incubating *L. plantarum* LysM-LacLMLreu- and LysM-LacZLbul-displaying cells in 50 mM NaPB (pH 6.5) at various temperatures (–20, 4, 30, 50 °C). At certain time intervals, samples were withdrawn, the residual activity was measured using *o*NPG as the substrate under standard assay conditions and the τ_1/2_ value was determined.

To test the reusability of immobilized enzymes, several repeated rounds of lactose conversion at 30 °C using LysM-LacLMLreu- and LysM-LacZLbul-displaying cells and at 50 °C using LysM-LacZLbul-displaying cells were carried out with 600 mM initial lactose in 50mM NaBP (pH 6.5) and constant agitation (500 rpm). The enzyme activity during these repeated cycles with intermediate two washing steps was measured using *o*NPG as the substrate under standard assay conditions.

### 4.13 Lactose Conversion and Formation of Galacto-Oligosaccharides (GOS)

The conversion of lactose was carried out in discontinuous mode using *L. plantarum* cells displaying β-galactosidase LacZ from *L. bulgaricus* (LysM-LacZLbul). The conversion was performed at 30 °C using 205 g/L initial lactose concentration in 50 mM NaPB (pH 6.5) and constant agitation (500 rpm). *L. plantarum* LysM-LacZLbul displaying cells were added to equivalent concentrations of 1.0 U_Lac_/mL of reaction mixture. Samples were withdrawn at intervals, heated at 99 °C for 5 min and further analyzed for lactose, galactose, glucose and GOS present in the samples.

### 4.14 Analysis of Carbohydrate Composition

The carbohydrate composition in the reaction mixture was analyzed by high-performance liquid chromatography (HPLC) equipped with a Dionex ICS-5000+ system (Thermo Fisher Scientific) consisting of an ICS-5000+ dual pump (DP) and an electrochemical detector (ED). Separations were performed at room temperature on CarboPac PA-1 column (4 × 250 mm) connected to a CarboPac PA-1 guard column (4 × 50 mm) (Thermo Fisher Scientific) with flow rate 1 mL/min. All eluents A (150 mM NaOH), B (150 mM NaOH and 500 mM sodium acetate) and C (deionized water) were degassed by flushing with helium for 30 min. Separation of D-glucose, D-galactose, lactose and allolactose was carried out with a run with the following gradient: 90% C with 10% A for 45 min at 1.0 mL/min, followed by 5 min with 100% B. The concentration of saccharides was calculated by interpolation from external standards. Total GOS concentration was calculated by subtraction of the quantified saccharides (lactose, glucose, galactose) from the initial lactose concentration. The GOS yield (%) was defined as the percentage of GOS produced in the samples compared to initial lactose.

### 4.15 Statistical Analysis

All experiments and measurements were conducted at least in duplicate, and the standard deviation (SD) was always less than 5%. The data are expressed as the mean ± SD when appropriate.

## 5 Conclusions

This work describes the immobilization of two lactobacillal β-galactosidases, a β-galactosidase from *L. reuteri* of the heterodimeric LacLM-type and one from *L. bulgaricus* of the homodimeric LacZ-type, on the *Lactobacillus* cell surface using a peptidoglycan-binding motif as an anchor, in this case, the single LysM domain Lp_3014 from *L. plantarum* WCFS1. The immobilized fusion LysM-β-galactosidases are catalytically efficient and can be reused for several repeated rounds of lactose conversion. Surface anchoring of β-galactosidases in *Lactobacillus* results in safe, non-GMO and stable biocatalysts that can be used in the applications for lactose conversion and production of prebiotic galacto-oligosaccharides.

## Figures and Tables

**Table 1:** β-Galactosidase activities in cell-free lysates of *E. coli* celIs carrying different expression vectors.

Expression Vector	Volumetric Activity (k·U/L Culture Medium)		Specific Activity (U/mg Protein)	

	Non-Induced	Induced	Non-Induced	Induced
pBAD3014LacLMLreu	n.d.	11.1 ± 1.5	n.d.	6.04 ± 0.03
pBAD3014LacZLbul	n.d.	46.9 ± 2.7	n.d.	41.1 ± 0.9

n.d.: not detected.

**Table 2:** Immobilization of **(A)** recombinant lactobacillal β-galactosidases on *L. plantarum* WCFS1 cell surface and **(B)** recombinant β-galactosidase from *L. bulgaricus* DSM 20081 (LysM-LacZLbul) on the cell surface of different *Lactobacillus* spp.

	Residual Activities in Supernatant	Immobilization Yield *a* (IY)	Activity on Cell Surface *b*		Activity Retention *c* (AR)	Amount of Active Surface Anchored β-gal *d*

	(%)	(%)	(%)	U/g DCW	(%)	mg/g DCW
**(A) Enzyme** (on *L. plantarum* WCFS1 cell surface)						
LysM-LacLMLreu	93.5 ± 1.2	6.53	3.06 ± 0.08	179 ± 5	46.9	0.99 ± 0.02
LysM-LacZLbul	68.1 ± 0.1	31.9	20.3 ± 0.2	1153 ± 12	63.5	4.61 ± 0.05

**(B) *Lactobacillus* spp.** (with enzyme LysM-LacZLbul)						
*L. plantarum* WCFS1	68.1 ± 0.1	31.9	20.3 ± 0.2	1153 ± 12	63.5	4.61 ± 0.05
*L. bulgaricus* DSM 20081	71.3 ± 0.9	28.7	14.0± 0.9	795 ± 53	48.5	3.18 ± 0.11
*L. casei*	76.1 ± 0.9	23.9	15.1 ± 0.8	861 ± 48	63.2	3.44 ± 0.09
*L. helveticus* DSM20075	75.3 ± 0.9	24.7	14.3 ± 0.5	812 ± 27	57.7	3.25 ± 0.11

a IY (%) was calculated by subtraction of the residual enzyme activity (%) in the supernatant after immobilization from the total activity applied (100%).

b Activity on the cell surface (%) is the percentage of enzyme activity measured on the cell surface to the total applied activity. Activity on the cell surface (U/g DCW) is calculated as the amount of enzyme (Units) per g dry cell weight.

c Activity retention, AR (%), is the ratio of activity on the cell surface (%) to IY (%).

d It was calculated based on specific activities of purified LacLMLreu of 180 U/mg protein [16] and of purified LacZLbul (His Tagged) of 250 U/mg protein [12]. Values given are the average value from at least two independent experiments, and the standard deviation was always less than 5%.

**Table 3:** Stability of *L. plantarum* β-galactosidase-displaying cells at various temperatures ^a^.

LysM-LacLMLreu		LysM-LacLZLbul	

Temperature	$\tau_{\frac{1}{2}}$	Temperature	$\tau_{\frac{1}{2}}$
–20 °C	6 months	–20 °C	6 months
4 °C	3 months	4 °C	Nd ^b^
30 °C	55 h	30 °C	120 h
50 °C	nd ^b^	50 °C	30 h

a *L. plantarum* galactosidase-displaying cells were incubated in 50mM sodium phosphate buffer (NaPB), pH 6.5 at different temperatures. Experiments were performed at least in duplicates.

b not determined.

**Table 4:** Strains and plasmids used in the study.

Strains or Plasmids	Relevant Characteristics	Reference Source
**Strains**		
*L. plantarum* WCFS1		[32]
*L. delbrueckii* subsp. *bulgaricus* DSM 20081		DSMZ
*L. casei*		BOKU
*L. helveticus* DSM 20075		DSMZ
*E. coli* HST08	Host strain	Clontech

**Plasmids**		
pBAD_3014_AgESAT_DC	Amp^r^; pBAD derivate with the LysM domain sequence from Lp3014 fused to the hybrid antigen AgESAT_DC	[44]
pBAD3014LacLMLreu	Amp^r^; pBAD_3014_AgESAT_DC derivative with a fragment of *lacLM* genes instead of the gene fragment encoding AgESAT_DC	This study
pBAD3014LacZLbul	Amp^r^; pBAD_3014_AgESAT_DC derivate with *lacZ* fragment instead of the gene fragment encoding AgESAT_DC	This study
pHA1032	Amp^r^; pET21d derivative for expression of *lacLM* from *L. reuteri* in *E. coli*	[16]
pTH103	Erm^r^; *spp*-based expression vector pSIP409 for expression of *lacZ* from *L. bulgaricus* DSM 20081 in *L. plantarum* WCFS1	[12]

**Table 5:** Primers used in the study.

Primer	Sequence* 5′→3′	Restriction Site Underlined
Fwd1LreuSalI	GAGTTCAACTGTCGAC*CAAGCAAATATAAA*	*Sal*I
Rev1LreuEcoRI	AGCCAAGCTTCGAATTC*TTATTTTGCATTC*	*EcoR*I
Fwd2LbulSalI	GTTCAACTGTCGAC*AGCAATAAGTTAGTAAAAGAAAAAAGAG*	*Sal*I
Rev2LbulEcoRI	CAGCCAAGCTTCGAATTC*TTATTTTAGTAAAAGGGGCTGAATC*	*EcoR*I

* The nucleotides in italics are the positions that anneal to the DNA of the target genes (*lacLM* or *lacZ*).
